# Peroxiredoxin-6 protects against oxidative stress-mediated hypertrophic remodeling in JPH2-A399S knock-in mice

**DOI:** 10.1016/j.ijcha.2026.102005

**Published:** 2026-09-03

**Authors:** Callum J. Quinn, Satadru K. Lahiri, Ann P. Quick, Sören Brandenburg, Dennis Uhlenkamp, Antrix Jain, Lucia M. Moreira, Sun Y. Jung, Svetlana Reilly, Rana Sayeed, George Krasopoulos, Stephan E. Lehnart, Xander H.T. Wehrens

**Affiliations:** a Cardiovascular Research Institute, USA; b Department of Integrative Physiology, Baylor College of Medicine, Houston, TX 77030, USA; c Department of Cardiology and Pneumology, Cellular Biophysics and Translational Cardiology Section, University Medical Center Göttingen, Georg-August-University, Göttingen D-37075, Germany; d Advanced Technology Cores, USA; e Division of Cardiovascular Medicine, Radcliffe Department of Medicine, British Heart Foundation Centre of Research Excellence, University of Oxford, John Radcliffe Hospital, Oxford OX3 9DU, UK; f Department of Biochemistry and Molecular Pharmacology, Baylor College of Medicine, Houston, TX 77030, USA; g Cardiothoracic Surgery, Oxford Heart Centre, John Radcliffe Hospital, Oxford OX3 9DU, UK; h Department of Medicine (in Cardiology), USA; i Department of Neuroscience, USA; j Department of Pediatrics (Division of Cardiology), USA; k Center for Space Medicine, Baylor College of Medicine, Houston, TX 77030, USA

**Keywords:** Junctophilin-2, Peroxiredoxin, Hypertrophic cardiomyopathy, Oxidative stress

## Abstract

**Background:**

Most mutations causing hypertrophic cardiomyopathy (HCM) affect sarcomeric proteins. Mutations in junctophilin-2 (JPH2) are also implicated, but the underlying mechanisms remain unclear. An A405S variant in JPH2 was identified in a male adolescent patient with interventricular septal (IVS) hypertrophy. The corresponding mouse variant (A399S) produces comparable IVS hypertrophy, establishing causality. Prior data indicated that altered intracellular Ca^2+^ handling is unlikely to be the primary driver.

**Methods:**

We generated a CRISPR knock-in mouse model carrying the JPH2-A399S variant. Co-immunoprecipitation mass spectrometry and STED nanoscopy were used to identify JPH2 binding partners. Reactive oxygen species (ROS) were assessed with dihydroethidium in isolated myocytes. Adeno-associated virus serotype 9 (AAV9) was employed to overexpress peroxiredoxin 6 (PRDX6) in mutant hearts.

**Results:**

PRDX6 was identified as a novel and abundant JPH2-interacting protein. PRDX6 expression was selectively downregulated in the IVS of JPH2-A399S mice and was also reduced in human failing hearts. JPH2-A399S mice exhibited increased ROS levels specifically in IVS myocytes. AAV9-mediated PRDX6 overexpression reversed the IVS hypertrophy phenotype.

**Conclusions:**

These findings identify PRDX6 downregulation and consequent oxidative stress as a key mechanism driving JPH2-A399S-associated HCM. The results reveal a previously unrecognized role for JPH2 in cardiometabolic regulation and suggest that restoring PRDX6 levels may represent a therapeutic strategy for this form of HCM.

## 1 Introduction

Hypertrophic cardiomyopathy (HCM) is the most common inherited cardiac disease and is characterized by left ventricular wall and interventricular septal hypertrophy, which predisposes patients to arrhythmia and sudden cardiac death [1]. Although the majority of HCM-causing mutations affect sarcomeric proteins, variants in non-sarcomeric proteins, including dyadic protein junctophilin-2 (JPH2), have also been implicated in HCM and other inherited cardiomyopathies [2]. JPH2 is essential for maintaining the structural integrity of cardiac dyads and plays a critical role in excitation-contraction coupling. Emerging evidence suggests that JPH2 may have additional functions beyond dyadic organization [3], [4].

An A405S variant in JPH2 was identified in a male adolescent patient presenting with marked basal septal hypertrophy [5]. We previously generated a pseudo knock-in model expressing the equivalent A399S variant on a cardiac-specific JPH2 knockdown background and demonstrated that these mice developed basal septal hypertrophy despite preserved total JPH2 levels [5]. Notably, these animals did not exhibit major alterations in intracellular Ca^2+^ handling [5]. More recently, we generated true A399S-JPH2 knock-in mice using CRISPR/Cas9 gene editing. These mice similarly developed interventricular septal (IVS) hypertrophy without changes in ventricular JPH2 expression [6]. Despite these observations, the molecular mechanisms linking the JPH2-A399S variant to hypertrophic remodeling remain incompletely understood.

In the present study, we used the CRISPR-generated JPH2-A399S knock-in mice to investigate potential alternative pathways. We identified peroxiredoxin 6 (PRDX6), a key antioxidant enzyme [7], as a novel binding partner of JPH2. We found that PRDX6 expression is selectively reduced in the IVS of JPH2-A399S mice and is also decreased in human failing hearts. Furthermore, JPH2-A399S mice exhibited increased reactive oxygen species (ROS) levels specifically in IVS myocytes. Importantly, AAV9-mediated overexpression of PRDX6 reversed the IVS hypertrophic phenotype. These findings suggest that the JPH2-A399S variant promotes HCM through dysregulation of PRDX6, leading to increased oxidative stress.

## 2 Methods

### 2.1 Animal studies

All animal procedures were conducted in accordance with the National Institutes of Health *Guide for the Care and Use of Laboratory Animals* and were approved by the Baylor College of Medicine Institutional Animal Care and Use Committee (IACUC). The JPH2-A399S knock-in mice were generated by knocking in the human nonsynonymous variant c.1213G > T via CRISPR/Cas9 embryo microinjections, as described [6]. All animals included in this study had a C57BL/6 J background. In total, 19 JPH2-A399S homozygous mice and 17 wildtype (WT) littermate controls were used with ages ranges from 3 to 5 months, which included an approximately equal number of males and female mice.

### 2.2 Human samples

Human myocardial tissue samples were obtained from the Oxford Cardiovascular Biobank; studies were approved by the South Central-Berkshire B Research Ethics Committee (REF: 18/SC/0404). Written informed consent was obtained from all participants in accordance with the Declaration of Helsinki. All samples were anonymized prior to analysis. A total of 8 patients were included in the study; all patients underwent cardiac surgery (coronary artery bypass and valve repair/replacement) in the John Radcliffe hospital, Oxford. Left ventricular biopsies were collected during cardiac surgery, before cardiopulmonary bypass and immediately processed for cell isolation (described below) or snap-frozen until use in subsequent experiments.

### 2.3 Co-Immunoprecipitation and mass spectrometry

Ventricular tissue lysates were pre-cleared by incubation with protein A/G agarose beads at 4 °C. After centrifugation, supernatants containing 40 μg of total protein were incubated overnight at 4 °C with either anti-JPH2 antibody or matched IgG control. Protein A/G beads were then added, and the mixture was agitated for 2 h at 4 °C. Beads were washed three times with dilution buffer, resuspended in NuPAGE LDS Sample Buffer, and heated at 70 °C. Proteins were separated by SDS-PAGE on 10% Bis-Tris NuPAGE gels (Life Technologies, Carlsbad, CA) and visualized with Coomassie Brilliant Blue staining.

Entire lanes were excised and subjected to in-gel trypsin digestion [8]. Peptides were analyzed by nano-LC-MS/MS using a nano-LC 1000 system (Thermo Fisher Scientific, San Jose, CA) coupled to an Orbitrap Elite mass spectrometer. Peptides were separated using a 70-min gradient of 4–26% acetonitrile containing 0.1% formic acid at a flow rate of 800 nL/min. Data were acquired in data-dependent mode (top 25) with MS1 scans at 120,000 resolution (375–1300 *m*/*z*) and MS2 scans in the ion trap (CID 29%). Raw data were searched against the mouse NCBI RefSeq database using Mascot (v2.4) [9] within Proteome Discoverer (v2.1). Precursor and fragment mass tolerances were set to 20 ppm and 0.5 Da, respectively. Dynamic modifications included oxidation (M), N-terminal acetylation, deamidation (N/Q), and destreak (C). Peptide-spectrum matches were validated at 5% FDR using Percolator [10], and protein quantification was performed using the label-free iBAQ approach via the gpGrouper algorithm.

### 2.4 Western blotting

Myocardial tissue was homogenized in lysis buffer containing protease inhibitors [6]. Equal quantities of protein were separated using SDS-PAGE and transferred to PVDF membranes. Membranes were blocked in 5% non-fat milk and incubated overnight with primary antibodies against PRDX6 (1:1000, ab59543, Abcam, Waltham, MA), GAPDH (1:2000 sc-398,125, Santa Cruz Biotechnology, Dallas, TX), or calsequestrin-2 (CSQ2; 1:2000, PA1–913 ThermoFisher Scientific, Waltham, MA). After incubation with appropriate secondary antibodies, bands were visualized and quantified using densitometry.

### 2.5 Immunofluorescence stimulated emission depletion (STED) nanoscopy

Cardiomyocytes were isolated from the interventricular septum of adult wild-type and JPH2-A399S mice using a modified Langendorff perfusion system. Hearts were perfused with Ca^2+^-free buffer (in mM: NaCl 120.4, KCl 14.7, KH_2_PO_4_ 0.6, Na_2_HPO_4_ 0.6, MgSO_4_ 1.2, HEPES 10, NaHCO_3_ 4.6, taurine 30, 2,3-butanedione-monoxime 10, glucose 5.5, pH 7.4 adjusted with NaOH) followed by collagenase type II digestion [11], [12]. Isolated cardiomyocytes were plated on laminin-coated coverslips, fixed in 4% paraformaldehyde for 5 min, and permeabilized/blocked for 2 h (10% bovine calf serum, 0.2% Triton in PBS). Cells were incubated overnight at 4 °C with primary antibodies against PRDX6 (1:500, ab59543, Abcam), JPH2 (1:500, sc-398,125, Santa Cruz Biotechnology), RyR2 (1:500, HPA-020028, Sigma-Aldrich), followed by incubation with STAR 635P or STAR 580 secondary antibodies (1:1000, Abberior, Goettingen, Germany). Samples were mounted in ProLong Gold antifade reagent (Thermo Fisher Scientific, Waltham, MA). Super-resolution imaging was performed using a Leica TCS SP8 STED microscope equipped with a 100×/1.40 oil immersion objective. Images were acquired with a pixel size of 16.23 × 16.23 nm and processed using ImageJ/Fiji (pixel dwell time 400 ns, scanning speed 600 Hz, 32× line averaging, white light laser excitation at 635 nm and 580 nm, and a 775 nm STED laser).

### 2.6 Quantification of reactive oxygen species using dihydroethidium (DHE)

Cardiac myocytes were freshly isolated from the left ventricular wall and interventricular septum of 4–5-month-old wild-type and JPH2-A399S mice [13]. Mice were anesthetized by isoflurane and euthanized by cervical dislocation. Hearts were rapidly excised and perfused via a Langendorff apparatus with Ca^2+^-free Tyrode's solution containing Liberase (22 μg/mL, Sigma-Aldrich, St. Louis, MO). Calcium was gradually reintroduced to a final concentration of 1.8 mM. Isolated myocytes were plated on laminin-coated coverslips and incubated with 20 μM DHE for 30 min at 37 °C. After washing, cells were imaged immediately using a Zeiss LSM 880 confocal microscope. DHE was excited at 514 nm and emission was collected at 680–720 nm. Mean fluorescence intensity was quantified using Fiji after background subtraction.

### 2.7 Echocardiography and AAV9-mediated PRDX6 overexpression

Transthoracic echocardiography was performed using a Vivo2110 imager (Fujifilm VisualSonics, Toronto, Canada) in 4-month-old wild-type and JPH2-A399S mice to assess cardiac structure and function, with particular attention to interventricular septal thickness in diastole (IVS;d) and left ventricular ejection fraction. A subset of JPH2-A399S mice received a single retro-orbital injection of 5.0 × 10^11^ genome copies of AAV9-TNT4-PRDX6-emGFP. Four weeks after injection, echocardiography was repeated to evaluate the effect of PRDX6 overexpression on septal thickness.

### 2.8 Statistical analysis

Data were analyzed using GraphPad Prism (version 11.0.2). Continuous normally distributed variables are presented as mean ± SEM and non-normally distributed variables are presented as median + IQR. Statistical significance was set at *P* < 0.05. The specific statistical tests used for each experiment (Mann-Whitney *U* test, two-way ANOVA followed by Fisher's LSD post-hoc test, or one-way ANOVA followed by Tukey's post-hoc test) are indicated in the corresponding figure legends.

## 3 Results

### 3.1 PRDX6 is a novel and abundant binding partner of JPH2 and is downregulated in JPH2-A399S mouse IVS and human failing hearts

**Fig. 1:**
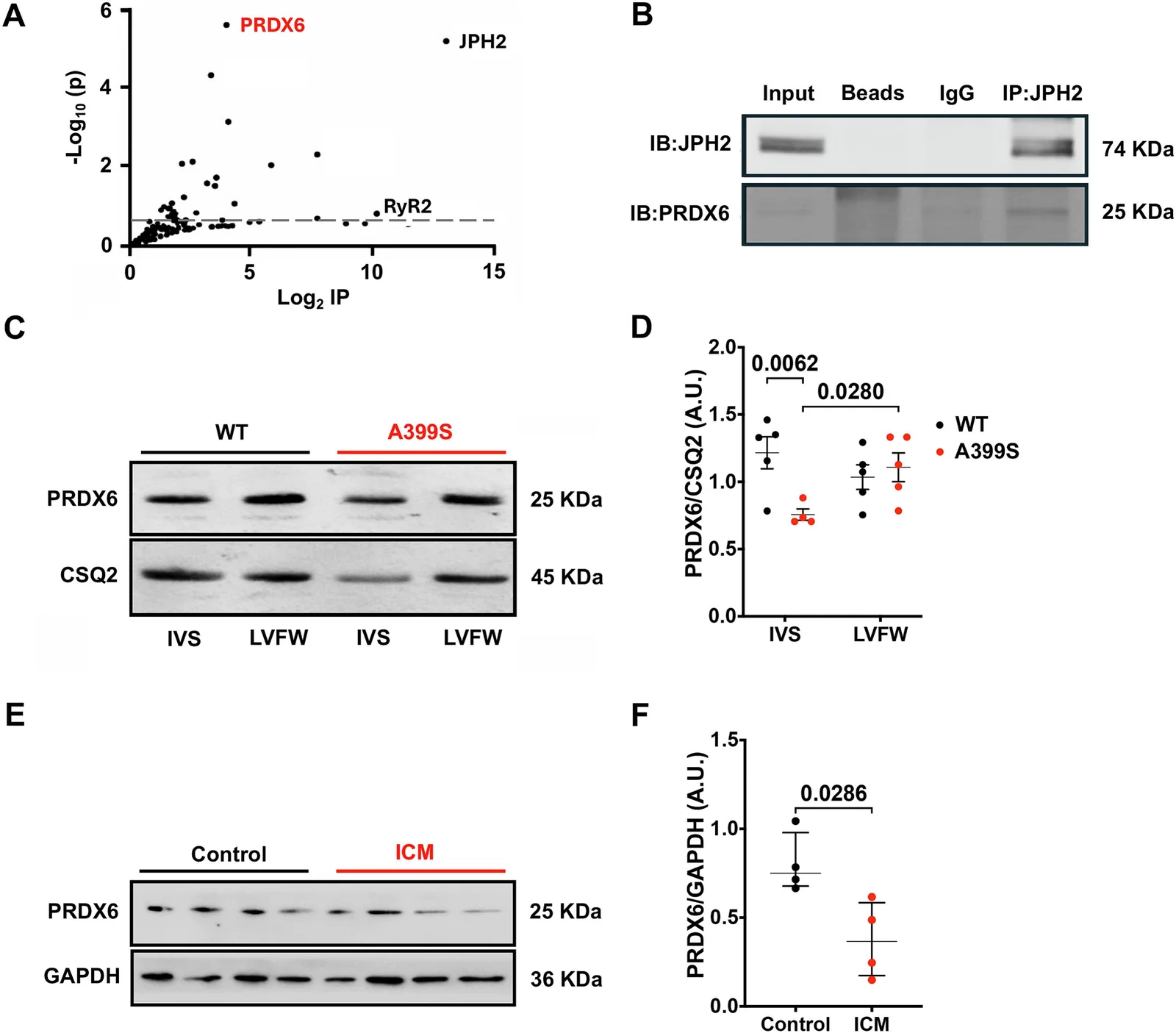
JPH2 interacts with PRDX6, which is downregulated in the IVS of JPH2-A399S mice and in human failing hearts. (**A**) Volcano plot of IP enrichment vs statistical significance from JPH2 co-immunoprecipitation mass spectrometry showing PRDX6 as one of the most abundant proteins in the JPH2 pulldown. RyR2 is shown as a positive control. The x-axis shows log_2_(IP enrichment), where IP represents the immunoprecipitation enrichment ratio. The y-axis shows -log_10_(p) using nominal *p*-values. Horizontal dashed line at -log_10_(0.05) = 1.3 indicates threshold corresponding to *p* < 0.05 (**B**) Co-immunoprecipitation immunoblot confirming the physical interaction between JPH2 and PRDX6. (**C, D**) Representative immunoblot (**C**) and quantification (**D**) of PRDX6 protein levels in the interventricular septum (IVS) and left ventricular free wall (LVFW) of wild-type (WT) and JPH2-A399S knock-in mice. PRDX6 abundance is selectively reduced in the IVS of A399S mice. *N* = 4–5 mice per group. Data analyzed by two-way ANOVA with Fisher's LSD post-hoc test. (**E, F**) Representative immunoblot (**E**) and quantification (**F**) of PRDX6 protein levels in left ventricular tissue from patients with ischemic cardiomyopathy (ICM) compared with non-failing control hearts. *N* = 4 samples per group. Data analyzed by the Mann-Whitney *U* test. CSQ2 and GAPDH were used as loading controls.

To identify novel proteins that may link the JPH2-A399S variant to hypertrophic remodeling, we performed co-immunoprecipitation mass spectrometry using ventricular tissue from JPH2-A399S mice. As expected, RyR2 was among the most highly enriched JPH2-interacting proteins, validating our experimental approach (Fig. 1A). Notably, the antioxidant enzyme peroxiredoxin 6 (PRDX6) was identified as one of the most significant JPH2-binding partners (Fig. 1A). This interaction was confirmed by co-immunoprecipitation followed by immunoblotting (Fig. 1B).

Western blot analysis revealed that PRDX6 protein levels were significantly lower in the interventricular septum (IVS) of JPH2-A399S mice compared with both the left ventricular free wall (LVFW) of the same animals and the IVS of wild-type (WT) littermates (Fig. 1C-D). Given the clinical overlap between hypertrophic cardiomyopathy and ischemic cardiomyopathy [14], we next examined PRDX6 expression in human myocardial samples. PRDX6 abundance was also significantly reduced in left ventricular tissue from patients with ischemic cardiomyopathy compared with non-failing control hearts (Fig. 1E-F). Collectively, these data demonstrate a previously unrecognized physical interaction between JPH2 and PRDX6 and show that PRDX6 downregulation occurs in both the region-specific hypertrophic phenotype of JPH2-A399S mice and in human heart failure.

### 3.2 JPH2 and PRDX6 exhibit nanoscale colocalization at the *Z*-line that is preserved in JPH2-A399S myocytes

**Fig. 2:**
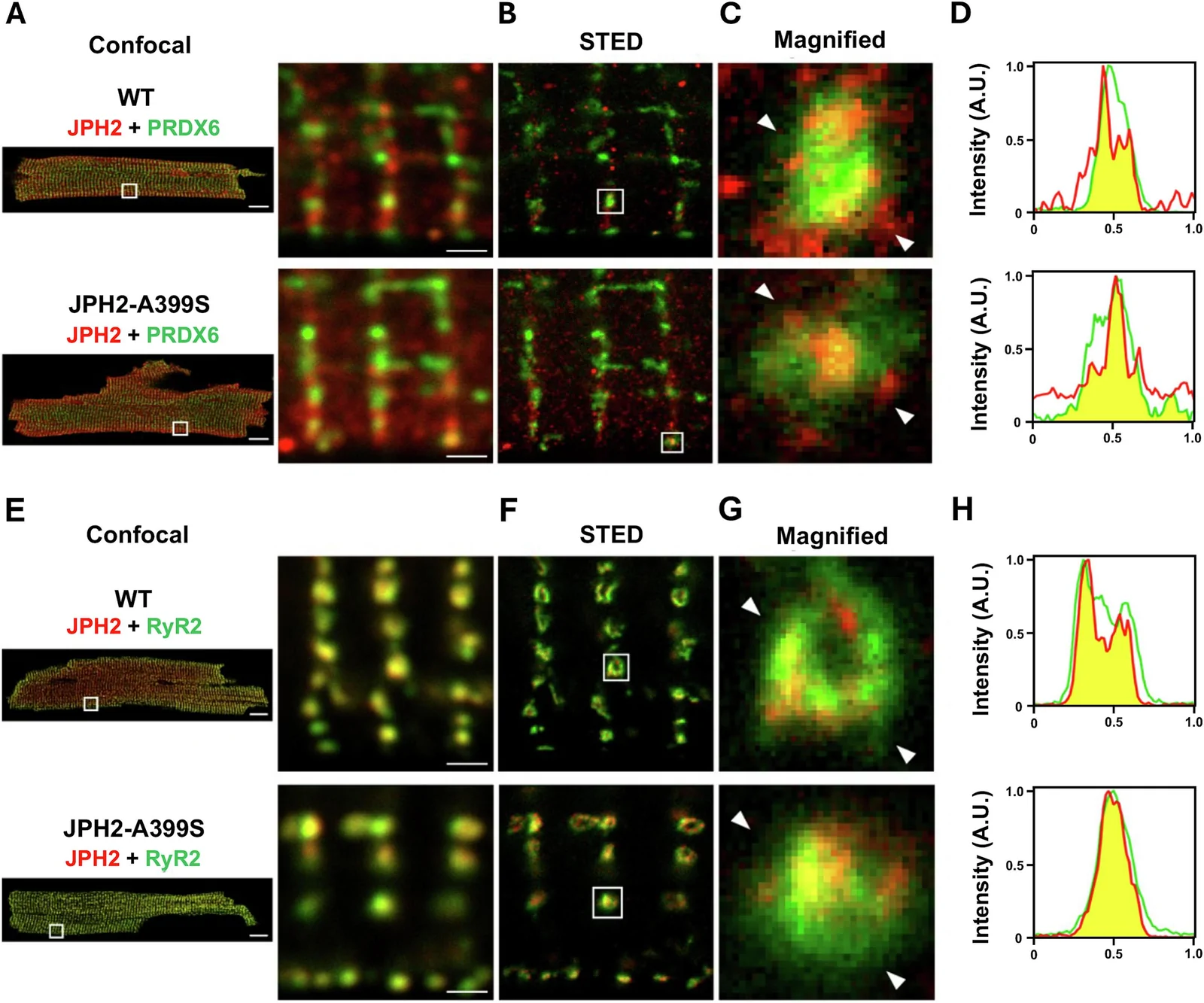
Nanoscale colocalization of JPH2 with PRDX6 and preserved JPH2-RyR2 organization in JPH2-A399S IVS cardiomyocytes. Both confocal and stimulated emission depletion (STED) super-resolution microscopy were used to examine the spatial relationship of JPH2 (red) with either PRDX6 (green; **A-D**) or RyR2 (green; ***E*-H**) in isolated IVS cardiomyocytes from WT and JPH2-A399S mice. Representative confocal images are shown next to corresponding STED images for the same region of interest. White boxes (**B, F**) indicate regions magnified in rightward images (**C, G**). White arrows highlight the diagonal line's direction used for signal intensity profiling. Line-scan intensity profiles illustrate the co-immunofluorescence intensity distributions, confirming the nanometric spatial association between the labeled protein pairs, each for JPH2/PRDX6 (**D**) and JPH2/RyR2 (**H**). Scale bars: 10 μm (confocal overview; **A, E**) and 1 μm (magnified confocal and corresponding STED panels **B, F**).

To characterize the spatial relationship between JPH2 and PRDX6, we performed immunofluorescence imaging using both confocal microscopy and stimulated emission depletion (STED) super-resolution nanoscopy in isolated IVS cardiomyocytes (Fig. 2A-D). Confocal imaging showed that both JPH2 and PRDX6 localize primarily to the Z-lines in WT and JPH2-A399S myocytes. STED imaging resolved finer structural details, revealing that PRDX6 forms smaller punctate structures frequently juxtaposed to or partially overlapping with larger JPH2 clusters (Fig. 2B and C). Line-scan intensity profiles (Fig. 2D) confirmed colocalization of PRDX6 with JPH2 at these sites. Although overall PRDX6 signal intensity was reduced in JPH2-A399S myocytes, the nanoscale spatial relationship between the remaining PRDX6 and JPH2 clusters remained qualitatively similar to that observed in WT cells.

### 3.3 JPH2-RyR2 organization is preserved in JPH2-A399S cardiac myocytes

We next assessed whether the JPH2-A399S variant disrupts JPH2-RyR2 clustering, given the established role of this interaction in excitation-contraction coupling. Both confocal and STED imaging demonstrated that the organization and colocalization of JPH2 with RyR2 were preserved in JPH2-A399S IVS myocytes compared with WT controls (Fig. 2E-H). These findings are consistent with our earlier observations that intracellular Ca^2+^ handling is largely unaffected in this model [5].

### 3.4 JPH2-A399S mice exhibit region-specific increases in oxidative stress

**Fig. 3:**
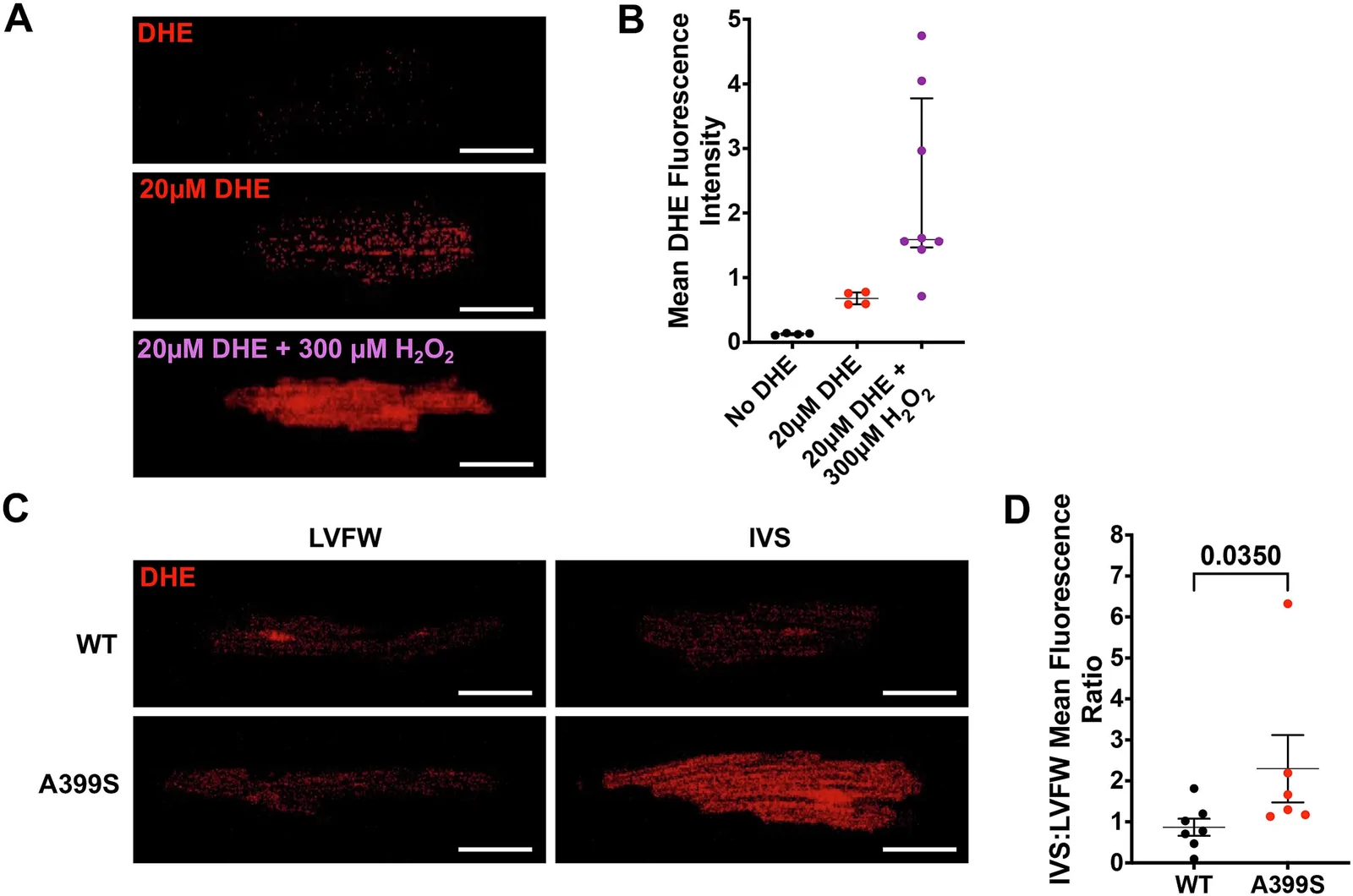
JPH2-A399S mice exhibit increased oxidative stress in the IVS. (**A, B**) Validation of the dihydroethidium (DHE) probe in WT cardiomyocytes. Representative confocal images (**A**) and quantification (**B**) of DHE fluorescence in cells treated with vehicle (no DHE), 20 μM DHE, or 20 μM DHE + 300 μM H_2_O_2_. *n* = 4–8 cells. (**C, D**) Representative DHE fluorescence images (**C**) and quantification of the IVS-to-LVFW fluorescence ratio (**D**) in freshly isolated cardiomyocytes from WT and JPH2-A399S mice. *N* = 6–7 mice per group **and** data in (**D**) was analyzed by Mann-Whitney *U* test.

Because PRDX6 functions as an antioxidant enzyme [15], we evaluated reactive oxygen species (ROS) levels in freshly isolated cardiomyocytes using dihydroethidium (DHE). Validation experiments confirmed that DHE fluorescence reliably increased in response to exogenous H_2_O_2_ (Fig. 3A-B). Due to variability in absolute DHE signal between cell preparations, we quantified fluorescence as the IVS-to-LVFW ratio within each heart. This ratio was significantly elevated in JPH2-A399S mice compared with WT littermates (Fig. 3C-D), indicating a selective increase in ROS within the IVS [5].

### 3.5 AAV9-mediated PRDX6 overexpression reverses IVS hypertrophy in JPH2-A399S mice

**Fig. 4:**
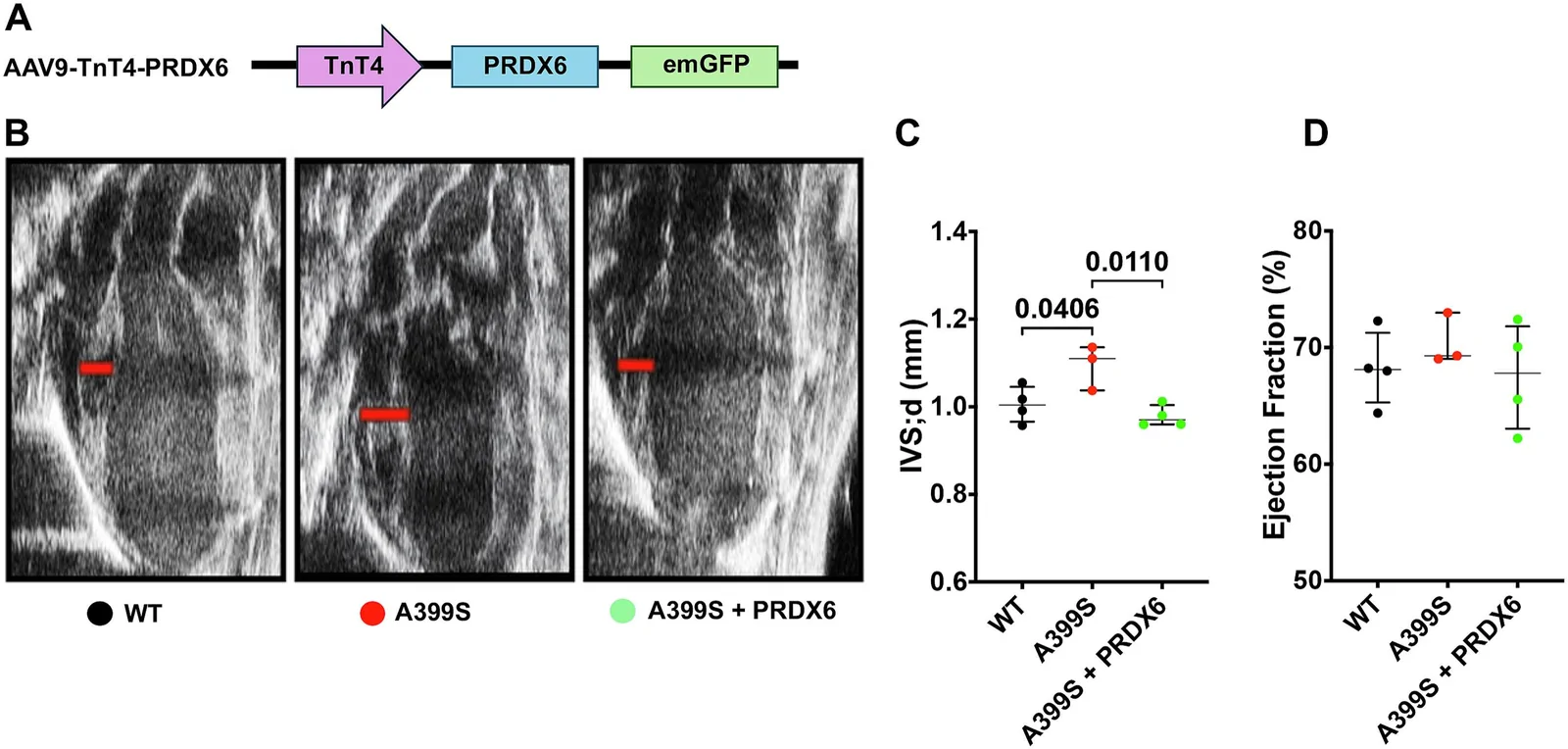
AAV9-mediated overexpression of PRDX6 rescues IVS hypertrophy in JPH2-A399S mice. (**A**) Schematic of the AAV9-TnT4-PRDX6-emGFP vector used for cardiac myocyte-specific overexpression of PRDX6. (**B**) Representative long-axis echocardiographic images from WT, JPH2-A399S, and JPH2-A399S mice treated with AAV9-PRDX6. Red bars indicate the interventricular septum (IVS). (**C, D**) Quantification of interventricular septal thickness in diastole (IVS;d) (**C**) and left ventricular ejection fraction (EF) (**D**). *N* = 3–4 mice per group. Data analyzed by one-way ANOVA.

To determine whether reduced PRDX6 expression contributes causally to the hypertrophic phenotype, we overexpressed PRDX6 in cardiac myocytes of JPH2-A399S mice using an AAV9 vector driven by the cardiac troponin T (TnT4) promoter (Fig. 4A). At 4 months of age, untreated JPH2-A399S mice displayed the expected increase in interventricular septal thickness in diastole (IVS;d) compared with WT controls (Fig. 4B-C). Echocardiography revealed that AAV9-PRDX6 administration prevented IVS hypertrophy (Fig. 4C). Left ventricular ejection fraction remained unchanged across all groups (Fig. 4D). These findings demonstrate that restoration of PRDX6 expression is sufficient to rescue the IVS-specific hypertrophic phenotype in JPH2-A399S mice.

## 4 Discussion

**Fig. 5:**
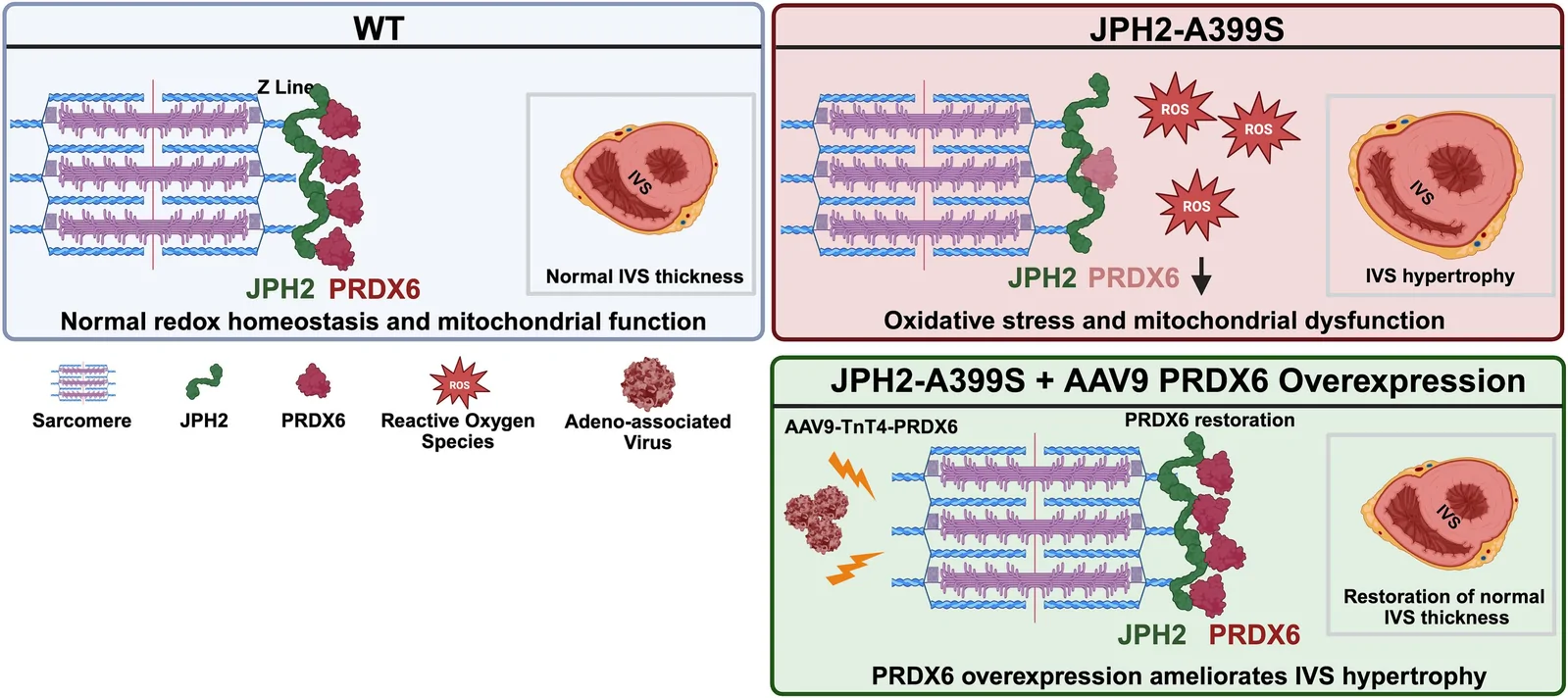
Proposed model: PRDX6 downregulation and oxidative stress link the JPH2-A399S variant to region-specific hypertrophic cardiomyopathy. Schematic illustration summarizing the key findings of the study. The JPH2-A399S mutation leads to selective reduction of PRDX6 in the IVS, resulting in increased ROS production, which drive IVS-specific hypertrophy. AAV9-mediated restoration of PRDX6 rescues the hypertrophic phenotype. Figure made using BioRender.

In this study, we used a CRISPR-generated JPH2-A399S knock-in mouse model to investigate the mechanisms underlying the interventricular septum (IVS)-specific hypertrophic cardiomyopathy (HCM) phenotype associated with the human JPH2-A405S variant [5]. Our findings reveal that the A399S mutation leads to downregulation of the antioxidant enzyme peroxiredoxin 6 (PRDX6), resulting in increased reactive oxygen species (ROS) production that selectively affects the IVS (Fig. 5). These changes appear to drive the hypertrophic remodeling observed in this model, as demonstrated by the ability of AAV9-mediated PRDX6 overexpression to rescue the IVS hypertrophy phenotype. The preferential involvement of the interventricular septum in this model mirrors the asymmetric septal hypertrophy frequently observed in clinical HCM, for which transapical beating-heart septal myectomy has emerged as an effective treatment option in obstructive cases [16].

To our knowledge, this is the first report of a physical interaction between JPH2 and PRDX6. Although PRDX6 has previously been shown to exert cardioprotective effects through its antioxidant and membrane-repair functions [17], [18], its role in HCM has not been previously described. We found that PRDX6 is selectively downregulated in the IVS of JPH2-A399S mice and is also reduced in human failing hearts. This region-specific loss of PRDX6 correlated with elevated ROS levels in IVS cardiomyocytes. These observations suggest that disruption of JPH2-PRDX6 signaling contributes to oxidative stress, providing a novel mechanistic link between a non-sarcomeric mutation and HCM pathogenesis.

Notably, JPH2-RyR2 clustering and organization remained intact in JPH2-A399S myocytes, consistent with our earlier findings of largely preserved Ca^2+^ handling in this model [5]. These results indicate that the hypertrophic phenotype driven by the A399S variant is unlikely to result primarily from disrupted excitation-contraction coupling, but rather from dysregulation of redox homeostasis.

The mechanisms underlying the selective downregulation of PRDX6 in the IVS, despite ubiquitous expression of the mutant JPH2 protein, remain unclear. This regional specificity suggests that additional local factors, such as differences in mechanical stress, metabolic demand, or transcriptional regulation, may influence PRDX6 stability or expression in the IVS. Future studies employing transcriptomic and proteomic approaches will be important to elucidate how the A399S mutation selectively impairs PRDX6 in this region.

Importantly, cardiac myocyte-specific overexpression of PRDX6 via AAV9 was sufficient to normalize IVS thickness in JPH2-A399S mice without affecting ejection fraction. This rescue experiment provides strong evidence that PRDX6 downregulation is a key driver of the hypertrophic phenotype and highlights the therapeutic potential of targeting oxidative stress or restoring PRDX6 levels in this form of HCM. These findings are complemented by recent reports on the efficacy and safety of mavacamten, a cardiac myosin inhibitor, in non-obstructive hypertrophy cardiomyopathy [19].

Finally, multiple trails using angiotensin-receptor blockers (ARBs) to treat HCM patients have generally not demonstrated consistent reductions in left ventricular mass or clear disease-modifying benefits [20]. Although, some positive observations have been made in early stage sarcomeric disease [21]. Our findings suggest a mechanistic explanation for why ARBs may be less effective in this genetic context. The oxidative stress we observe is driven by selective, region-specific downregulation of PRDX6 secondary to the JPH2-A399S variant, rather than by the classic angiotensin-II/AT1R-NADPH-oxidase axis that ARBs target [22]. Because the proximal lesion is loss of a key antioxidant enzyme that physically interacts with JPH2, simply interrupting up stream Ang II would not be expected to restore PRDX6 levels fully or correct redox imbalance in the interventricular septum. This distinction underscores that oxidative stress in HCM is heterogeneous and genotype dependent. Therapies that restore the specific missing antioxidant capacity (as demonstrated by PRDX6 overexpression) may therefore be more effective for JPH2-linked disease than non-specific RAAS blockade.

## 5 Conclusion and future directions

In summary, we have identified a novel JPH2-PRDX6 signaling axis that links the A399S/A405S variant to region-specific hypertrophic remodeling through increased oxidative stress. These findings expand our understanding of JPH2 beyond its canonical role in excitation-contraction coupling and reveal a previously unrecognized contribution of redox imbalance to non-sarcomeric HCM [23]. The successful rescue of the phenotype by PRDX6 overexpression suggests that strategies aimed at restoring antioxidant capacity may offer therapeutic benefit for patients carrying this and potentially other JPH2 variants. Similar efforts to characterize distinct phenotypes, such as those in severe atrial cardiomyopathy, highlight the heterogeneity of cardiac remodeling processes [24].

Future studies exploring the regulation of this pathway and its relevance to other cardiomyopathies will be important to fully realize its translational potential. For example, the specific role of PRDX6 in the JPH2-A399S HCM phenotype is supported by the multiple experimental observations described here. However, to strengthen this causal claim, future experiments should utilize pharmacological or genetic loss-of-function approaches to specifically block the PRDX6 pathway. These may include PRDX6 inhibitors, dominant-negative mutants, or conditional knockouts on the JPH2-A399S background.

Furthermore, the rescue of septal hypertrophy by PRDX6 overexpression demonstrates a targeted antioxidant intervention that directly addresses the observed molecular defect. Future investigations may wish to perform additional antioxidant challenges to determine whether ROS inhibition (using *N*-acetylcysteine, tempol or other ROS scavengers) is therapeutically sufficient or whether the bifunctional activities of PRDX6 (peroxidase and PLA_2_) are required for the reversal of the JPH2-A399S HCM phenotype.

### 5.1 Study limitations

A limitation of our study relates to our AAV9-mediated PRXD6 overexpression experiments. Specifically, the lack of a concurrent vehicle or empty vector control group. The selective rescue of the interventricular-septal phenotype in combination with the region-specific reduction of endogenous PRDX6 and elevated ROS in untreated mutant hearts strongly supports the specificity of the AAV9-mediated intervention. However, future studies incorporating an empty AAV9 control will be required to exclude non-specific vector effects.

## CRediT authorship contribution statement

**Callum J. Quinn:** Writing – original draft, Visualization, Investigation, Formal analysis. **Satadru K. Lahiri:** Writing – review & editing, Investigation, Formal analysis, Conceptualization. **Ann P. Quick:** Writing – review & editing. **Sören Brandenburg:** Writing – review & editing, Methodology, Investigation. **Dennis Uhlenkamp:** Writing – review & editing, Investigation. **Antrix Jain:** Writing – review & editing, Investigation, Formal analysis. **Lucia M. Moreira:** Writing – review & editing, Methodology, Investigation. **Sun Y. Jung:** Writing – review & editing, Investigation, Formal analysis. **Svetlana Reilly:** Writing – review & editing, Resources. **Rana Sayeed:** Resources. **George Krasopoulos:** Resources. **Stephan E. Lehnart:** Writing – review & editing, Supervision, Methodology, Funding acquisition, Conceptualization. **Xander H.T. Wehrens:** Writing – review & editing, Supervision, Project administration, Methodology, Conceptualization.

## Funding

This work was supported by 10.13039/100000968American Heart Association Innovative Project Award grant 25IPA1457115 to SKL. Human studies were supported by the 10.13039/501100000274British Heart Foundation (BHF) Senior (FS/SBSRF/22/31033) Fellowships to S.R. STED nanoscopy and data analysis was supported by Germany's Excellence Strategy - EXC 2067/1-390729940 Multiscale Bioimaging to S.E.L.

## Declaration of competing interest

The authors declare the following financial interests/personal relationships which may be considered as potential competing interests: Dr. Wehrens is a consultant for Rocket Pharmaceuticals. All other authors declare that they have no known competing financial interests.

## Data Availability

The data that support the findings of this study are available from the corresponding author upon reasonable request.
